# Electronic health record implementation and healthcare workers’ work characteristics and autonomous motivation—a before-and-after study

**DOI:** 10.1186/s12911-022-01858-x

**Published:** 2022-05-03

**Authors:** Gepke L. Veenstra, Eric F. Rietzschel, Eric Molleman, Erik Heineman, Jan Pols, Gera A. Welker

**Affiliations:** 1 Department of Surgery, University Medical Center Groningen, University of Groningen, Hanzeplein 1, Huispostcode LA10, 9713 GZ Groningen, The Netherlands; 2 Department of Psychology, University of Groningen, Groningen, The Netherlands; 3 Department of Human Resource Management and Organizational Behavior, Faculty of Economics and Business, University of Groningen, Groningen, The Netherlands; 4 Center for Educational Development and Research in Health Professions, University Medical Center Groningen, University of Groningen, Groningen, The Netherlands; 5 UMC Staff Policy and Management Support, University Medical Center Groningen, University of Groningen, Groningen, The Netherlands

**Keywords:** Healthcare workers, Work motivation, Work characteristics, Before-and-after study, Electronic health record, EHR implementation

## Abstract

**Background:**

Technological innovation in healthcare is often assumed to contribute to the quality of care. However, the question how technology implementation impacts healthcare workers has received little empirical attention. This study investigates the consequences of Electronic Health Record (EHR) implementation for healthcare workers’ autonomous work motivation. These effects are further hypothesized to be mediated by changes in perceived work characteristics (job autonomy and interdependence). Additionally, a moderating effect of profession on the relationship between EHR implementation and work characteristics is explored.

**Methods:**

A quantitative uncontrolled before-and-after study was performed among employees from a large university medical centre in the Netherlands. Data were analysed following the component approach for testing a first stage moderated mediation model, using Generalized Estimating Equations (GEE).

**Results:**

A total of 456 healthcare workers (75 physicians, 154 nurses, 145 allied healthcare professionals, and 82 administrative workers) finished both the baseline and the follow-up survey. After EHR implementation, perceived job autonomy decreased, whereas interdependence increased. In line with our hypothesis, job autonomy was positively associated with autonomous motivation. In contrast to our expectations, interdependence also showed a positive association with autonomous motivation. Autonomous motivation was stable over the course of EHR implementation. This study did not provide support for a moderating effect of profession: no differences were observed between the various professions regarding the changes in their experienced job autonomy and interdependence after EHR implementation.

**Conclusions:**

Our study showed that healthcare professionals’ perceptions of their work characteristics, but not their autonomous motivation, were changed after EHR implementation, and that these experiences were relatively similar for physicians, nurses, and allied healthcare professionals. The stability of healthcare workers’ autonomous motivation may be explained by the opposite effects of decreased job autonomy and increased interdependence, and by the EHR being in line with healthcare workers’ values. The changes in job autonomy and interdependence may have consequences beyond motivation, for example by affecting clinical decision-making, proactive behaviour, and the quality of teamwork. These potential consequences of EHR implementation warrant further research.

**Supplementary Information:**

The online version contains supplementary material available at 10.1186/s12911-022-01858-x.

## Background

Healthcare workers face constant changes, due to technological and medical innovations that aim to improve the quality, efficiency, or safety of care [1, 2]. The factors contributing to (or hindering) the implementation of these innovations have been widely investigated, showing—among other things—that the involvement of staff is essential for the success of these innovations [1, 3, 4]. However, one largely under-investigated area within and outside healthcare is how these innovations impact workers in terms of their work motivation and the way they experience their work [2, 3, 5–7].

The lack of knowledge about the consequences of innovations for healthcare workers is problematic, because work motivation and work characteristics are important predictors of performance and wellbeing [8, 9]. More specifically, autonomous forms of motivation among healthcare workers have been associated with higher quality and safety of care, and with lower susceptibility for burnout [10, 11]. Furthermore, healthcare workers’ motivation and their work characteristics affect the extent to which they proactively learn from positive and adverse incidents and speak up in the face of threats to patient safety, which is important for continuous quality improvement and organizational learning within the complex healthcare setting [12, 13].

In other words, hospital workers are a valuable resource of high-quality and sustainable care and their work characteristics and motivation are important determinants of their performance in clinical practice [10, 14]. However, the consequences of technology implementation for healthcare workers are largely unknown [2, 3, 5–7]. The aim of the present study, therefore, is to examine the impact of the implementation of technology on the work characteristics and autonomous motivation of healthcare workers. The possible changes in work characteristics and motivation are investigated in an uncontrolled before-and-after study within the context of an organizational change. This organizational change entails an innovation that many healthcare institutions have implemented during the past decades, namely an *electronic health record* (EHR).

With this study, we aim to make three contributions to the literature. First, we aim to shed light on the ‘complex array of forces’ [15, p. 15], consisting of work characteristics, and individual, technological, and organizational factors, that affect the success and outcomes for workers after technology implementation. We aim to do this by investigating the effects of EHR implementation on healthcare workers’ work characteristics and their subsequent work motivation. Thereby, we answer calls for research on the impact of technology implementation in the work setting [6, 15]. Second, we aim to go beyond existing work on technology implementation, which tends to focus on specific groups of workers [5, 15, 16]. However, the consequences of technology implementation may vary for different workers, for example due to higher versus lower levels of job autonomy [15]. Therefore, this study involves four major groups of healthcare workers (physicians, nurses, allied healthcare professionals, and administrative workers) and explores possible differences in their reactions to EHR implementation. Finally, we aim to contribute to the literature on the self-determination theory of motivation by following three recommendations to advance the theory, namely: (a) we investigate the relationships between concrete work characteristics and motivation, (b) we do this in the context of technology implementation, and (c) we use a longitudinal (before-and-after) design to do this [6].

**Fig. 1:**
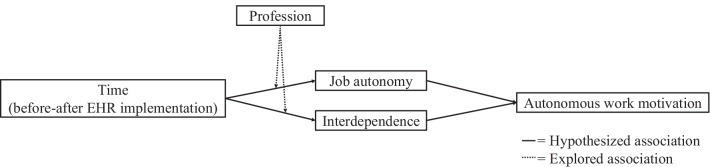
Theoretical framework of this study

In short, we are curious about the relationship between EHR implementation and healthcare workers’ autonomous work motivation, and we anticipate that two work characteristics, namely job autonomy and interdependence, mediate this relationship. In addition, we explore the moderating effect of profession on the relationship between EHR implementation and work characteristics. The theoretical framework of this study is depicted in Fig. 1. In the following, we will first describe EHRs and explain how they may affect work motivation, followed by an explanation of how we believe work characteristics will mediate this relationship. Then, we will address the possible moderating role of profession.

### Electronic health records

An EHR is a longitudinal digital record of a patient’s health information, such as demographical information, medical history, diagnoses, medications, radiology images, laboratory data, healthcare workers’ notes and other clinically relevant information [17]. Although an EHR could be considered the digital equivalent of the classic paper or computer-based health record of a patient, it differs from the traditional record in important ways. Most notably, an EHR is an *integrated* record, containing information from all healthcare workers involved in a patient’s care. This information can be accessed instantly and securely by authorized users [17, 18]. Compared to the traditional records they replace, EHRs are expected to increase the availability and quality of information, and to support information exchange among health care providers [16, 17]. Additionally, EHRs are often equipped with tools to improve healthcare practice by stimulating healthcare workers’ adherence to guidelines and organizational protocols (for example, through reminders and by blocking access or orders where necessary). Therefore, EHRs have many anticipated functional benefits, such as improved quality, safety, and cost-effectiveness of care, and enhanced clinical decision-making [14, 17, 18].

Importantly, in order to harvest these potential functional benefits, clinical and operational changes must be made [19]. Thereby, EHRs may change and standardize workflows and documentation requirements, shift tasks from one healthcare worker to another, and affect communication during the provision of patient care [14, 17–21]. For example, Taylor et al. found that after EHR implementation, face-to-face communication between doctors and nurses was significantly reduced, which was associated with lower agreement between these workers about the care plan for their patients [22]. This is just one illustration of how the implementation of an EHR changes the nature of work for healthcare workers. Moreover, these changes probably alter the work characteristics of healthcare workers, which in turn are likely to affect their work motivation [2].

### Work motivation

Work motivation is defined as ‘a set of energetic forces that originate both within as well as beyond an individual’s being, to initiate work-related behaviour, and to determine its form, direction, intensity, and duration’ [23, p 23]. One widely used theory of motivation and the way it is affected by the (work) environment is the self-determination theory (SDT) [8]. The SDT describes a continuum of various types of motivation, ranging (simply put) from *not wanting* to do something (amotivation), through *having to* do something (controlled or extrinsic motivation), to *wanting* to do something (autonomous or intrinsic motivation) [8]. In the current study, we focus on autonomous motivation, as research has shown that an engaged and autonomously motivated healthcare workforce is essential for the delivery of high-quality care [10, 24, 25].

Autonomous work motivation refers to doing something out of reasons stemming from within oneself; i.e., it stems from a sense of self-determination. Autonomous motivation is defined as wanting to put effort into the work because employees find their work enjoyable or interesting (intrinsic motivation), or because the work is in line with their values, personal goals, and identity (identified regulation) [8, 26].

Further, the SDT states that motivation becomes more autonomous when workers experience satisfaction of their basic psychological needs for autonomy (the need to feel in control), relatedness (the need to maintain positive relationships with others), and competence (the need to experience a sense of mastery) [8, 9]. Importantly, the SDT states that *work characteristics* hold the potential to boost or thwart autonomous motivation, as they affect the extent to which workers experience satisfaction of their basic psychological needs [8, 9, 27]. Specifically, autonomous motivation is thought to be boosted (vs. thwarted) by work characteristics that support (vs. undermine) employees’ sense of autonomy, competence, and relatedness. Based on this premise, we anticipate that EHR implementation will be associated with changes in workers’ autonomous motivation through EHR-related changes in healthcare workers’ work characteristics [6, 15].

### Work characteristics

Work characteristics, defined as ‘the attributes of the task, job, and social and organizational environment’ [28, p 28], and their consequences for motivation and performance, have been widely investigated [2, 28]. A vast body of research shows that work characteristics, such as job autonomy, clarity about roles, task variety, feedback, team climate, and leadership style, can significantly boost or weaken motivation [2, 28, 29].

Because, as explained above, EHR implementation requires several clinical and operational changes [5, 19], healthcare workers’ work characteristics are likely to be affected by these changes [15]. In the following paragraphs, we will argue that two specific work characteristics are particularly relevant in case of EHR implementation, and are especially likely to affect satisfaction of the basic psychological needs for autonomy and relatedness: *job autonomy* and *interdependence*. Note that, in this study, we do not consider the need for competence [8], as we anticipate that feelings of competence are more likely to be affected by the quality of training, IT skills and ‘teething troubles’ of the system [7, 19, 30], which are not the main focus of this study.

#### Job autonomy

Job autonomy is defined as the extent to which the job allows workers freedom to plan their work, to make decisions and to choose work methods [28, 29]. Although the behaviour of healthcare workers is partly directed by protocols and regulations, autonomy is ingrained in the jobs of workers who provide direct patient care (e.g., physicians, nurses, or allied healthcare professionals), since their jobs require them to act upon their specific professional knowledge and skills [31, 32]. A high level of autonomy allows flexibility, which supports healthcare workers’ clinical decision-making [33]. In addition to supporting the provision of patient care, job autonomy gives healthcare workers a sense of volition at work. This feeling of being self-controlled satisfies their need for autonomy, which contributes to their autonomous motivation [8, 27].

Previous research has shown that technology-related and other organizational changes have the potential to alter employees’ work characteristics [15, 28, 34]. We argue that EHRs are likely to affect healthcare workers’ job autonomy, as they enable external control over workers’ clinical decision-making and scheduling of work [15, 35]. For example, EHRs often have standardized methods of record keeping, specific built-in workflows that are based on standardized work processes, and may be equipped with decision support tools that guide healthcare workers’ decision-making process for routine tasks [18]. Furthermore, formal control of work procedures may be increased by defining role-based access and role-based permissions (e.g., only physicians, rather than other staff members, are allowed to order specific tests or medications), and adherence to workflows may be stimulated through reminders or other actions of the system, thereby reducing the extent to which healthcare workers can freely organize their own work tasks [7, 19]. Previous research on EHR implementation found that primary care physicians experienced less autonomy after EHR implementation due to work scheduling interference [35], and nurses participating in a study by Bergey et al. [16, p 16] even referred to their hospital’s EHR as ‘a needy baby that has to be answered every time it cries’ to express their workflow-related experiences, thereby indicating reduced freedom to plan their tasks.

Based on these previous findings, we have formulated the following hypotheses:

##### Hypothesis 1a

the introduction of an EHR will be associated with a decrease in healthcare workers’ perceptions of job autonomy;

##### Hypothesis 1b

perceptions of job autonomy will be positively related to autonomous motivation;

##### Hypothesis 1c

taken together, EHR implementation will be negatively related to healthcare workers’ autonomous motivation through (i.e., mediated by) their perceived job autonomy.

#### Interdependence

The second work characteristic that we consider in this study is interdependence, which is defined as the ‘connectedness’ people experience in their job: the extent to which workers depend on others and others depend on them to complete their work [28]. In most healthcare settings, workers are highly dependent on each other when caring for and treating patients [5, 36]. When jobs are highly interdependent, healthcare workers need to mutually adjust and coordinate their efforts to realize high-quality care. Especially when these mutual adjustments require face-to-face interactions, there will be ample opportunities to develop relationships with others, and the extent to which these relationships are positive or negative will affect the fulfilment of the need for relatedness [28, 37]. According to the SDT, satisfaction (vs. dissatisfaction) of the need for relatedness will be positively (vs. negatively) associated with workers’ levels of autonomous motivation, respectively [8, 27].

However, the introduction of an EHR means that these mutual adjustments become much more controlled by the digital system; moreover, this will be accompanied by the standardization of operating procedures, so that work practices of different health care workers become tightly coupled and more interdependent [7, 16, 19, 38]. For example, in the hospital setting, the EHR may require the surgeon to place an order in the system for patient transfer from the operating room to the intensive care unit (ICU). Before EHR implementation, ICU workers could immediately start providing care, whereas after EHR implementation, they may have to await the surgeon’s order before being able to access the patient record and start providing care. This example illustrates previous observations from the literature that interdependence may lead to production blocking and process losses because employees have to wait for the input of others [39]. In such cases, interdependence may even lead to conflicts [40] that further impair positive interactions [41]. Thus, the implementation of an EHR may force new, and often undesirable, kinds of interdependence on healthcare workers. Furthermore, the need for face-to-face interactions likely lessens after EHR implementation [1, 19, 22, 42], which may decrease the opportunities for employees to develop positive social relationships [28, 43]. This is problematic, because high-quality interpersonal relationships are especially important for collaboration in highly interdependent work settings [44]. Moreover, less positive relationships lower the level of satisfaction of the need for relatedness, which will have a negative influence on autonomous work motivation [8, 9, 27].

Following the arguments above, we have formulated the following hypotheses:

##### Hypothesis 2a

the introduction of an EHR will be associated with an increase in healthcare workers’ perceptions of interdependence;

##### Hypothesis 2b

perceptions of interdependence will be negatively related to autonomous motivation;

##### Hypothesis 2c

taken together, EHR implementation is negatively related to healthcare workers’ autonomous motivation through (i.e., mediated by) perceived interdependence.

### Professional differences

In addition to these hypotheses, we also aim to explore an additional factor: the role of professional differences. The healthcare workforce in hospitals is diverse, being constituted by workers from various occupational backgrounds, including nurses, physicians, physician assistants, social workers, dieticians, and administrative workers. Physicians, nurses, and allied healthcare professionals (HCPs) are highly trained and socialized within their profession [5, 45, 46] and complex (in)formal hierarchies exist amongst these different professions, in which roles and responsibilities depend on one’s position in the hierarchy [16, 47]. This diversity might be reflected in people’s responses to the implementation of an EHR [5, 15, 47], because the distinct roles and methods of socialization amongst healthcare workers are likely to cause them to value and experience their work context and the EHR differently. For example, physicians traditionally hold a highly autonomous, self-regulating role [35]. It is possible that due to their traditionally high levels of autonomy, they value autonomy more than the other professions, and changes in autonomy may therefore be more salient to them [5, 7]. Nurses, as another example, have a key role in the clinical setting and to execute their work well, their relationships with others are essential [5]. Accordingly, research shows that they value the social aspect of their job highly [44, 48]. Therefore, changes to the interdependence of healthcare workers’ jobs as a consequence of EHR implementation may be most salient to nurses, compared to the other professions.

It should be noted that we do not expect the implementation of an EHR to change the professional *identities* of these different groups; we expect these to be relatively stable as a consequence of the high level of training and intense socialisation that healthcare professionals undergo when entering their profession [5, 45, 46, 49]. However, roles and hierarchies within healthcare may be affected by digitalization [5, 16, 50], and we argue that the implementation of an EHR may be associated with different *responses* for different professional groups, due to differences in how they experience their work context. There is little existing knowledge about professional differences regarding the consequences of technology implementation to build upon. Therefore, rather than formulating directional hypotheses, this study explores the moderating role of profession on the relationship between EHR implementation and work characteristics. In other words, we will explore whether any differences exist between four professional groups of healthcare workers (nurses, physicians, allied HCPs, and administrative workers) regarding the changes in their levels of autonomy and interdependence after EHR implementation.

## Methods

### Setting and procedure

The study took place in a large academic medical centre in the Netherlands. The data were collected in two waves: a baseline measure, taking place between September and the 1st of December 2017, and a follow-up measure, conducted between June and September 2018.^1^ The implementation of the EHR (Epic) took place on the 2nd of December 2017. Thus, all employees had (after receiving training before implementation) been working with the EHR for some months at the time of the second measurement. The survey was sent to all employees of the academic medical centre who were registered as working with the EHR; this included clinical (e.g., nurses and physicians) as well as non-clinical (e.g., administrative) staff. Employees who were registered in the organization as working with the EHR received an email with an invitation to participate in the survey, that contained a link to an online survey platform (Qualtrics). After giving informed consent, employees completed the measures described below. The survey ended with demographic questions regarding respondents’ age, gender, profession, tenure, and education. A total of three reminders to participate were sent to non-responders.

### Measures

#### Autonomous work motivation

Autonomous work motivation was measured with the six items constituting the identified and intrinsic motivation subscales from the Multidimensional Work Motivation Scale, which was validated in Dutch [51]. Example items are ‘*I have fun doing my job*’ and ‘*Putting effort into this job aligns with my personal values’*, α_baseline_ = 0.90; α_follow-up_ = 0.93).

#### Work characteristics

Perceptions of job autonomy and interdependence were measured using the Dutch version of the Work Design Questionnaire (WDQ-NL) [28, 52, 53].

Job autonomy was measured in terms of work scheduling and decision-making autonomy (6 items, e.g., ‘*The job allows me to make a lot of decisions on my own’*, α_baseline_ = 0.91; α_follow-up_ = 0.93). Interdependence was measured using six items (e.g., ‘*My job cannot be done unless others do their work*’, α_baseline_ = 0.86; α_follow-up_ = 0.85). All the items mentioned above were answered on a 7-point Likert Scale (1 = *completely disagree* to 7 = *completely agree*).

### Statistical analysis

Data were analysed using SPSS for Windows, version 23. Healthcare workers who were not physicians, nurses, allied HCPs, or administrative workers were excluded from analysis as this group of healthcare workers (including, among others, students, managers, lab workers, and EHR support workers) was deemed too diverse to draw meaningful conclusions. Participants who did not respond to the motivation scales (the main outcome variable) were considered to have provided incomplete responses and also excluded from analysis.

The outcome variable, autonomous motivation, was not normally distributed (with Shapiro–Wilk for baseline *W*(454) = 0.91, *p* < 0.01 and for follow-up *W*(454) = 0.87, *p* < 0.01). Therefore, Spearman’s rho was calculated to determine the correlations between the variables. Preliminary analyses included a non-parametric Kruskal–Wallis test to explore the differences between the professions regarding their levels of autonomous motivation, and for job autonomy and interdependence; an ANOVA was done for this purpose.

The Generalized Estimating Equations (GEE) method was used to test the hypotheses [54]. This approach to estimating the parameters of a generalized linear model assumes neither a normal distribution nor independent data, which makes it suitable for non-normally distributed data from repeated measurements. Additionally, the GEE method overcomes the issue of incorrect estimation of regression model parameters that may occur when the data consist of repeated measures, as it takes the correlation among responses given by the same participant into account. This requires specification of the correlation structure, which was set to unstructured (meaning that the correlation structure emerges from the data), which is appropriate for a within- and between subjects repeated measures design [54, 55].

The theoretical framework was tested following the ‘component’ approach, which entails performing joint-significance tests of multiple parameter estimates within the theoretical framework [56, 57]. In this approach, simple mediation is established by following recommendations that build upon the work by Baron and Kenny [58]. It involves the following steps of examining the parameter estimates representing the relationships (1) between the independent variable X (time) and the outcome variable Y (work motivation), (2) between the independent variable X (time) and the mediating variable(s) M (work characteristics), and (3) between M and Y, while controlling for X (i.e., time and work characteristics are simultaneous predictors of motivation). The effect is considered to be mediated (indirect) when the individual coefficients linking X to M and M to Y are both statistically significant (or neither of the 95% confidence intervals in step 1 and 2 includes zero), and the coefficient of X in step 3 is significantly smaller than in step 1. Further, moderated mediation is demonstrated when 4) there is a significant effect of the moderator variable Z (profession) on at least one path in the causal process linking X (time) to Y (motivation) via M (work characteristics), while 5) the remaining unmoderated path is not equal to zero (i.e., the 95% confidence intervals of the parameter estimates that represent the association between work characteristics and motivation do not include zero) [56, 57]. All hypothesis tests presented are two-sided, with alpha = 0.05 level tests, and 95% confidence intervals are given for each of the parameter estimates. As previous research shows that older employees might respond differently to EHR implementation than younger employees [19], we controlled for age in all regression analyses.

## Results

### Preliminary analysis

**Fig. 2:**
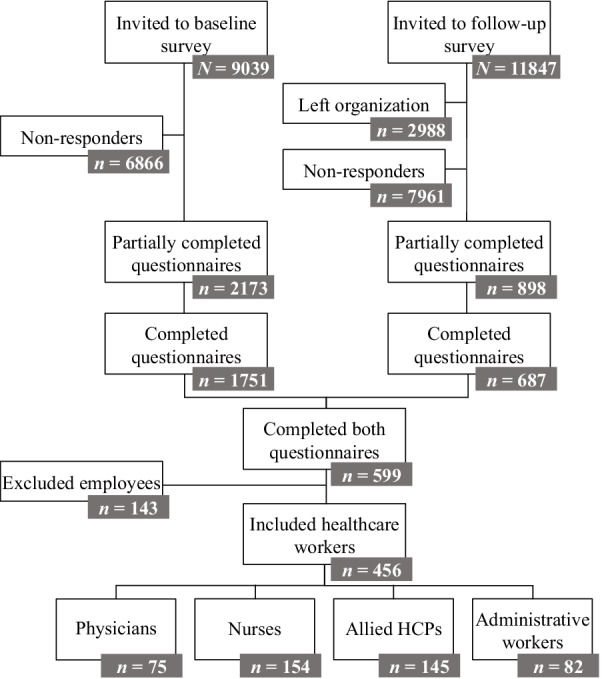
Flowchart of response and attrition

At baseline, the survey was (partially) completed by 2173 out of 9039 healthcare workers (a 24% response rate), and at follow-up, 898 out of 8859 healthcare workers still working at the same university medical centre (partially) completed the survey (a 10% response rate). A total of 599 participants completed both the baseline and the follow-up survey, yielding a 27.5% response rate relative to the baseline respondents. Of those, 456 participants belonged to the professional groups of interest, yielding our final sample. The flow diagram of response and attrition is depicted in Fig. 2.

The mean age of the included healthcare workers was 46.36 (*SD* = 11.13); the majority (79.4%) of participants identified as female, 20.0% identified as male and 0.7% identified as ‘other’ (e.g., non-binary). Of the study participants, 16% were physicians, 34% were nurses, 32% were allied HCPs (e.g., physician assistants or dieticians), and 18% were administrative workers (e.g., medical secretaries or financial administration department employees). The average job tenure of the healthcare workers was 11.81 (*SD* = 10.02) years.

The means, standard deviations and correlations among the variables of interest at baseline and follow-up are given in Table 1, showing that healthcare workers had relatively high autonomous motivation at both times (*M*_baseline_ = 6.16, *M*_follow-up_ = 6.15). The descriptive statistics of the study variables per profession at both measurements are given in Appendix Table 1. There were no differences between the various professions regarding their level of autonomous motivation at baseline (*X*^*2*^ (3) = 1.22, *p* = 0.75), nor at follow-up (*X*^*2*^ (3) = 1.21, *p* = 0.75). There were some differences between the professions regarding job autonomy (*F*_baseline_ (3, 452) = 5.46, *p* < 0.01; *F*_follow-up_ (3, 450) = 8.50, *p* < 0.01), which was lowest at both measurements amongst allied HCPs, compared to physicians, nurses, and administrative personnel. At both measurements, interdependence was highest amongst physicians and administrative workers, compared to nurses and allied HCPs (*F*_baseline_ (3, 452) = 9.48, *p* < 0.01; *F*_follow-up_ (3, 452) = 6.95, *p* < 0.01).

**Table 1:** Descriptive statistics—means (*M*), standard deviations (*SD*) and Spearman’s correlations of the measures of this study (*n* = 456)

	*M*	*SD*	1	2	3	4	5	6	7
Baseline									
1. Age	46.36	11.13	–						
2. Autonomous motivation	6.16	0.68	.02	**–**					
3. Job autonomy	4.86	1.18	.10*	.23**	–				
4. Interdependence	4.66	1.10	− .09*	.11*	.00	–			
Follow-up									
5. Autonomous motivation	6.15	0.79	.06	.61**	.16**	− .03	–		
6. Job autonomy	4.78	1.28	.11*	.21**	.74**	− .02	.23**	–	
7. Interdependence	4.88	1.08	− .11*	.06	− .11*	.59**	.05	− .01	–

Variables 2 to 7 were measured on a 7-point Likert scale

*p < .05; **p < .01 (2-tailed)

### Attrition analysis

At both measurements, the sample of healthcare workers participating in the study was representative of the occupational distribution of personnel working with the EHR in the academic medical centre. Women were slightly overrepresented in the final sample of health care workers who participated in both measurement moments, compared to the percentage of women working in the academic medical centre (79.4% (*n* = 456) versus 69.8% (*n* = 12,735; based on the year report of the academic medical centre), *X*^*2*^ (1) = 19.37, *p* < 0.01). No differences were found in the occupational distribution when comparing the group of respondents (i.e., the physicians, nurses, allied HCPs, and administrative workers, who completed both measurement moments (*N* = 456) with the baseline-only-respondents (*N* = 854). An independent samples t-test showed that the respondents who completed both measurements were somewhat older than the baseline-only-respondents (*M* = 46.36 versus *M* = 44.02, *t* (1308) = 3.6, *p* < 0.01). Although age might affect EHR responses, the consequences of self-selection were probably limited as no differences were found between the two groups with regard to any of the variables of interest.

### Hypothesis testing

In accordance with the component approach, we report on the relationships between time and each work characteristic, on the relationship between the work characteristics and autonomous motivation, and on the mediating effect of the work characteristic on the relationship between time and autonomous motivation. The results of the GEE analyses are presented in Table 2.

**Table 2:** Results of hypothesis testing: parameter estimates with confidence intervals (CIs) of the GEE regression analyses^a^

Step		Work characteristics				Autonomous motivation	
		Job autonomy		Interdependence			
		*β*	95% CI	*β*	95% CI	*β*	95% CI
1	Time					− .01	− .07 to .05
	Age					.01	− .01 to .01
	Intercept					6.10**	5.85 to 6.35
2	Time	− .09*	− .17 to − .01	.24**	.14 to .33		
	Age	.01^+^	.00 to .02	− .01*	− .02 to − .01		
	Intercept	4.46**	4.04 to 4.88	5.09**	4.74 to 5.44		
3	Time					− .01	− .08 to .05
	Job autonomy					.12**	.07 to .17
	Interdependence					.07**	.02 to .11
	Age					.01	− .01 to .01
	Intercept					5.23**	4.78 to 5.68
4	Time	− .10	− .30 to .09	.06	− .12 to .24		
	Nurses	− .02	− .30 to .26	− .71**	− .97 to − .46		
	Allied HCPs	− .33*	− .65 to − .01	− .65**	− .94 to − .36		
	Administrators	.19	− .16 to .54	− .25	− .58 to .07		
	Time × Nurses	.03	− .20 to .26	.20^+^	− .03 to .43		
	Time × Allied HCPs	− .04	− .29 to .22	.21	− .05 to .47		
	Time × Administrators	.11	− .15 to .37	.21	− .11 to .53		
	Age	.01^+^	.01 to .02	.01**	− .02 to − .01		
	Intercept	4.55**	4.07 to 5.02	5.63**	5.22 to 6.04		
5	Time					.01	− .16 to .18
	Job autonomy					.13**	.08 to .17
	Interdependence					.08**	.03 to .13
	Nurses					.11	− .07 to .29
	Allied HCPs					.10	− .08 to .28
	Administrators					− .08	− .29 to .14
	Time × Nurses					− .04	− .23 to .15
	Time × Allied HCPs					.01	− .19 to .21
	Time × Administrators					− .07	− .28 to .15
	Age					.00	− .01 to .01
	Intercept					5.08**	4.60 to 5.56

The reference category for time was baseline and for profession was physicians. *N* = 456

^a^To assure that performing multiple statistical tests did not lead to false positives, we also analysed the data on our (long) dataset using the much recommended PROCESS macro by Hayes, where time entered to the model as a binary predictor [60]. This resulted in regression coefficients very similar to those presented in Table 2, but it should be noted that the macro was not developed for repeated measures

^+^*p* < .10; *p < .05; **p < .01.

#### Job autonomy

As predicted by Hypotheses 1a and 1b, perceptions of job autonomy decreased after EHR implementation (*β* = − 0.09, *p* = 0.04) and perceived autonomy was positively associated with autonomous motivation (*β* = 0.12, *p* < 0.01). However, the mediation test showed that motivation neither decreased after EHR implementation (*β* = − 0.01, *p* = 0.74) in step 1, nor in step 3 of the mediation analysis. Therefore, not all the conditions that demonstrate mediation were met and we have to conclude that the data do not support Hypothesis 1c.

#### Interdependence

Furthermore, as predicted by Hypothesis 2a, healthcare workers’ perceptions of interdependence increased after EHR implementation (*β* = 0.24, *p* < 0.01). In contrast to Hypothesis 2b, which anticipated a negative effect of interdependence on autonomous motivation, our results actually showed a *positive* effect of interdependence on autonomous motivation (*β* = 0.07, *p* < 0.01). However, the conditions to confirm a mediation effect as formulated in Hypothesis 2c were not met, because autonomous motivation remained relatively stable over the course of time (*β* = − 0.01, *p* = 0.74) in both step 1 and in step 3 of the mediation analysis.

#### Profession

The interaction between time and profession was not statistically significant for autonomy (*X*^*2*^ (3, *N* = 452) = 1.53, *p* = 0.67), nor for interdependence (*X*^*2*^ (3, *N* = 452) = 3.75, *p* = 0.29), thereby failing to support a moderating effect of profession.

## Discussion

In this study, we examined the consequences of technology implementation, in this case EHR implementation, for healthcare workers’ autonomous motivation. We tested these consequences in a before-and-after field study among healthcare workers in an academic hospital, assessing participants’ perceived work characteristics both prior to and after the implementation of an EHR. Our aims for this study were threefold; we will discuss these three aims below.

### The consequences of EHR implementation

Our first aim was to shed light on the impact of technology implementation in the work setting, by investigating its consequences for autonomous motivation, and by addressing the mediating effects of job autonomy and interdependence. In line with previous research, we found that respondents’ levels of perceived job autonomy dropped slightly after EHR implementation, while job autonomy in turn was positively associated with autonomous motivation [16, 19, 27, 35].

As expected, perceptions of interdependence increased after EHR implementation; however, unexpectedly, perceived interdependence was positively (rather than negatively) associated with autonomous motivation. In our hypothesis we reasoned that the kind of interdependence that would increase after EHR implementation would diminish feelings of relatedness, as the system would lessen face-to-face contact, while at the same time making people more dependent on colleagues they had fewer interactions with, thereby potentially obstructing collaboration and interfering with positive interactions [1, 19, 22, 42]. Possibly, the clinical work still allowed for sufficient (informal face-to-face) contact to contribute to positive relationships at work. It could also be that the EHR facilitated rather than thwarted the coordination of interdependent tasks, thereby supporting collaboration and feelings of relatedness, which could explain the positive association between increased interdependence and autonomous motivation. Another explanation is that increased interdependence may have affected motivation in other ways, such as through an increased sense of responsibility, thereby contributing to internal work motivation [29]. For example, in a study among police officers, it was found that interdependence in teams of police officers was associated with a higher sense of responsibility for other people’s work, which contributed to the motivating potential of their jobs [59]. Likewise, Kuvaas [43] found that task interdependence among public sector employees was positively associated with intrinsic motivation. It appears, then, that the relation between interdependence and autonomous motivation is more complex and multifaceted than we initially hypothesized. Future research could address the dual nature of interdependence more extensively, for example by measuring the quality of relationships or by testing the mediating roles of a sense of responsibility versus a sense of relatedness.

Another unexpected finding was that healthcare workers’ autonomous motivation remained stable in the face of EHR implementation, despite decreased job autonomy and increased interdependence. This can be explained by the fact that the two indirect pathways (i.e., the effects of time through both mediators, one of which was positive, and the other negative) cancelled each other out [60]. More specifically, whereas the loss of autonomy may have decreased healthcare workers’ levels of autonomous motivation, interdependence was positively associated with autonomous motivation, and therefore its increase might have led to a boost of autonomous motivation, resulting in a net effect on motivation that approximates zero.

Yet another explanation for the stability of autonomous motivation may be that EHRs have a variety of motivation-relevant effects beside the changes in work characteristics analysed in the current paper. On the one hand, the EHR supports healthcare workers in achieving their work-related goals and values, but on the other hand the administrative burden resulting from an EHR may impair autonomous motivation [14, 20, 61, 62]. More specifically, the instrumental value of the EHR is likely to contribute to healthcare workers’ sense of identified regulation. Throughout the literature, it has been observed repeatedly that healthcare workers perceive the EHR as a vehicle to achieve quality and safety in patient care, which is one of their core values and as such supports their autonomous motivation [19, 27, 63]. Thus, healthcare workers generally endorse the idea of an EHR, which raises the question whether EHR implementation should actually be associated with an *increase* in autonomous motivation. However, as EHR systems were originally developed to support billing, administration, and regulation of healthcare [64], healthcare workers often experience practical struggles with the system and frustrations about usability issues and a lack of compatibility between the EHRs and their clinical work [19, 30, 45]. Because the quality of their clinical work is so important for healthcare workers, the struggles and frustrations that follow from working with these systems in clinical practice might negatively affect their intrinsic motivation, and might counteract the positive consequences of EHR implementation on autonomous motivation through an increased sense of identified regulation. We therefore recommend investigating whether the development of an EHR that is *primarily* designed to support patient care holds the potential to boost healthcare workers’ autonomous motivation.

### Professional differences in responses to EHR implementation

The second aim of our study was to contribute to the literature by exploring the moderating role of profession in healthcare workers’ reactions to EHR implementation. We found that the change over time was the same for all healthcare workers, despite initial differences between the professions regarding their levels of perceived job autonomy and interdependence, which suggests that the implementation of an EHR affected the work characteristics of all healthcare workers more or less equally. Above, we have argued that roles and hierarchies amongst the various workers might change as a consequence of innovations such as EHR implementation, but our findings point out that these longstanding traditions are unlikely to be radically affected by one innovation in healthcare. After all, healthcare professions (including the roles and hierarchies amongst them) are grounded in centuries-old institutions [5, 45, 46, 49], and, although identities might change and develop over time [5, 16, 50, 65], it is unlikely that the introduction of an EHR itself will alter professional identities, roles, and hierarchies substantially [46, 49]. Nonetheless, it has been argued that the combination of current developments in healthcare does affect the medical professions, whether it be by affecting the way in which they perform their work as a consequence of identifying with their profession, or the rise of new forms of professionalism, such as connective professionalism [46, 49]. The latter, the understanding of professionalism as a relational phenomenon, might also explain why task interdependence was positively rather than negatively associated with autonomous motivation. The changing meaning of professionalism and identification processes in the face of technological innovations in healthcare warrant more research.

Another explanation for the absence of a moderating effect of profession might be related to the use of self-reported measures of job autonomy and interdependence: the degree to which people value job autonomy and interdependence may be reflected in the extent to which people perceive these work characteristics to be present in the work environment. In other words, a physician and a member of administrative staff reporting a ‘moderately high’ level of autonomy may not report the same objective levels of autonomy, because their expectations and beliefs about what constitutes ‘high’ or ‘low’ autonomy in their respective jobs may differ. Therefore, the self-reported measures may have not been sensitive enough to distinguish valuing a specific work characteristic from actually experiencing that work characteristic. However, it could also be argued that perceived work characteristics are particularly important when it comes to predicting motivational outcomes: having an objectively high level of autonomy will not be motivating if it is not perceived as such, a topic which has been debated within SDT [66, 67]. Future research could further explore this issue by applying qualitative methods or using, where possible, more objective or implicit measures of work characteristics.

### Contributions to SDT

Finally, our third aim was to contribute to the literature on SDT by examining the relationship between concrete work characteristics and motivation in a longitudinal study, in addition to expanding the knowledge about the motivational consequences of technology implementation, as suggested by Deci et al. [6]. Our research sheds light on the relationships that job autonomy and interdependence have with autonomous motivation, suggesting that simultaneous changes in these work characteristics may counteract their respective consequences for work motivation. Given the potential roles of experienced responsibility and instrumentality of the EHR (as argued above), it could be relevant to look into the possibility of a fourth need as a predictor of work motivation, namely the need for beneficence (the sense of making a positive contribution) [68].

Another finding relevant to SDT is the stability of autonomous motivation in the face of technology-related organizational change. We have argued above that different aspects of the work environment or different types of motivation may have counteracted each other’s consequences, thereby illustrating that autonomous work motivation is the consequence of many facets of one’s job. Being such a broad construct, autonomous work motivation might be relatively resilient to fluctuations in the face of organizational change. This might be especially the case for healthcare workers and public professionals, who generally identify strongly with the goals and values that underlie their jobs, and who therefore have high levels of autonomous motivation [69]. In most cases of technology implementation, the goals and values of the jobs do not change, and may actually be more supported by the technology (even if the technology itself may be a hindrance). Therefore, it might be useful in future research to consider more specific forms of motivation (e.g., daily fluctuations in motivation or task motivation) to investigate the consequences of technology implementation. In addition, future researchers may want to explore whether technology implementation in a non-public setting, where autonomous motivation is less high [69], has different consequences for workers’ motivation.

### Strengths, limitations, and directions for future research

The strengths of this study encompass testing of the direct relationships between work characteristics and work motivation in the context of technology implementation and its before-and-after design, in addition to the contributions to the literature as described above. However, due to the setting and design of the study, the internal and external validity of our findings are open for discussion. Since our findings are based on the situation in one academic medical centre, their generalizability to other organizations and countries is inevitably limited. Furthermore, our research took place during the so-called ‘shakedown phase’ after EHR implementation (the period between 6 and 12 months after EHR implementation) [7, 19], which limits the generalizability of our findings to longer-term consequences. It is possible that the changes in workflow and care delivery after a major and complex infrastructure upgrade are only really achieved years after implementation. Therefore, it is possible that some of our effects (or the absence thereof) are due to the relatively short time that employees had had to work with the new system [14, 19]. Then again, other research that studies similar interventions reports similar time intervals (e.g., [7, 14, 38, 61]). Nevertheless, future research should address this by incorporating more follow-up measurements when evaluating the consequences of organizational change [70].

Furthermore, the correlational design of this study and the absence of a control group prevent us from drawing causal conclusions as about the consequences of the implementation of the EHR, since they might be attributable to confounding variables, such as local or national developments (e.g., work pressure due to staff shortages). The use of self-report questionnaires may have led to social desirability response bias, despite our efforts to encourage the participants to answer the survey accurately and honestly (e.g., by ensuring anonymity, stressing the importance of honest responses, and using validated questionnaires). Additionally, common method bias may have caused inflation of relationships between variables, and the low response rate (although comparable to similar longitudinal studies, [71]) may have resulted from some self-selection bias. It could be that autonomous motivation was found to be stable due to self-selection of participants, because participants who completed both questionnaires were highly autonomously motivated for their jobs. In contrast, participants whose motivation suffered after EHR implementation may not have completed the second questionnaire. The observed high level of autonomous motivation among participants may even have been associated with constructive coping styles during organizational change [72, 73], thereby preventing participants from negative outcomes. Future studies could investigate the moderating role of coping styles or try to overcome self-selection by participants.

This study enables us to point out several directions for future research. First, although the linearity of our theoretical framework enabled us to zoom in on some aspects of healthcare workers’ work experiences, the consequences of organizational change might have been more multidirectional than presented in our theoretical framework, as illustrated by the example about the coping strategies above. Second, while this study focused on two specific work characteristics as mediators, EHR implementation also results in other changes, for example an increase in the administrative burden or the substitution of interpersonal contact (with colleagues and patients) with information exchange in the EHR [1, 19, 22, 42]. Thus, future studies on the topic could incorporate more work characteristics, such as social support or work characteristics that likely affect satisfaction of the need for competence (e.g., the EHR may influence feedback loops and learning processes that may have an impact on the fulfilment of the need for competence). Likewise, future work could focus on other outcomes, such as controlled work motivation, the quality of teamwork or the work climate [1, 6, 42]. Third, as Priestman et al. found that the capacity to work with EHRs may be higher for junior staff compared to senior staff [19], we have controlled for age in our statistical analyses. Although we found that age was minimally associated with the outcomes of interest, a further exploration of the differences between junior and senior healthcare workers’ responses to technology implementation could provide an interesting avenue for future research. These topics could also be addressed by applying qualitative methods to explore aspects beside those presented in our framework and to explore the complexity of these constructs and the relationships between them.

### Practical implications

Practitioners who read this paper might conclude that technology implementation, such as an EHR, has few consequences for healthcare workers, as it does not appear to affect their motivation. Although we found autonomous motivation to be stable in the face of technology related organizational change, this conclusion is would be premature, as we observed that job autonomy and interdependence were both affected by EHR implementation. As such, our findings illustrate the importance of looking beyond autonomous motivation as an outcome. Work characteristics are not only important because of their possible effects on autonomous motivation [26]. For example, job autonomy is also crucial for making decisions that are in the best interest of patients and indispensable for continuous quality improvement, as it enables workers to act proactively and respond appropriately in the face of unanticipated events [10, 25, 32, 33]. Whereas technology, such as the EHR, might be useful to enforce organizational policy [35], it is important to realize that in a complex and unpredictable setting such as healthcare, there is a limit to prescribing professional conduct through policy [12]. It is therefore recommended that technology implementation should support healthcare workers’ autonomy. In this way, a balance can be established between keeping up quality via policy versus enabling healthcare workers to act upon their autonomous motivation to provide and improve quality of care.

Another point to consider when implementing new technologies is how this will affect teamwork and relationships among healthcare workers. As our study shows, interdependence might possibly be strengthened through the use of technologies. However, our study does not shed light on how EHRs affect the quality of the relationships among team members, and it is important to realize that EHRs may negatively affect mutual trust among team members because of ‘the tendency to conflate data entry with face-to-face communication’ [1, p. 1], and may thwart shared awareness due to information overload [42]. When implementing technologies, it is therefore important to consider its consequences for teamwork and the facilitation of mutual trust and shared awareness, which supports the delivery of high-quality patient care by health care teams.

## Conclusions

In conclusion, we found that implementation of an EHR was associated with a drop in perceived autonomy and an increase in perceived interdependence. Although this did not carry over into a change in autonomous motivation, these findings do warrant further research into the impact of technology implementation on the quality of teamwork in hospitals and the relationships between work characteristics and basic psychological need satisfaction, e.g., between interdependence and experienced relatedness, specifically. One key implication of this study is that there should be attention for healthcare workers’ job autonomy and the quality of their teamwork in the face of technology implementation, as these work characteristics not only affect their work motivation, but are also essential for the delivery of high-quality and safe patient care.

More generally, although it is impossible to predict future developments, it seems highly likely that the digitalization of healthcare will continue for a long time. In other words, healthcare workers will continuously be confronted with changes in the way they do their jobs and the way their work (as well as the way they work together) is organized. Research on how this affects them, their perceptions of their work, and their wellbeing, therefore remains crucial.

## Supplementary Information

**Additional file 1.** Descriptive statistics of the study variables per profession

## Data Availability

The quantitative data are not publicly available to guarantee the anonymity of our participants. The output of the PROCESS macro (see note Table 2) is available upon request from the corresponding author.

## Abbreviations

EHR: Electronic Health Record
GEE: Generalized Estimating Equations
HCP: Healthcare professional
SDT: Self-determination theory

## Footnotes

1 Only 5% of the respondents completed the survey later than 30 days after the first invitation for the follow-up questionnaire. Therefore, the length of use of the system was approximately equal for most (95%) of our respondents.
