# Supplementary material for: Electronic health record implementation and healthcare workers’ work characteristics and autonomous motivation—a before-and-after study
Source: BMC Med Inform Decis Mak. 2022 May 3;22:120. doi: 10.1186/s12911-022-01858-x (PMC9063104; doi:10.1186/s12911-022-01858-x)
Supplement: Supplementary file 1 — Additional file 1. Descriptive statistics of the study variables per profession [file 12911_2022_1858_MOESM1_ESM.docx]

| Additional File 1. Descriptive statistics of the study variables per profession | | | | | | | |
| --- | --- | --- | --- | --- | --- | --- | --- |
|  |  | Autonomous motivation  Mean rank | | Job autonomy  Mean (SD) | | Interdependence  Mean (SD) | |
|  | *n* | Baseline | Follow-up | Baseline | Follow-up | Baseline | Follow-up |
| Physicians | 75 | 224 | 217 | 4.93 (1.06) | 4.84 (1.17) | 5.15 (0.93) | 5.20 (0.90) |
| Nurses | 154 | 237 | 233 | 4.91 (0.96) | 4.84 (1.09) | 4.45 (0.93) | 4.70 (0.92) |
| Allied HCPs | 145 | 227 | 234 | 4.61 (1.38) | 4.48 (1.40) | 4.50 (1.23) | 4.75 (1.22) |
| Administrative staff | 82 | 219 | 221 | 5.14 (1.21) | 5.16 (1.36) | 4.87 (1.10) | 5.13 (1.14) |
